# Diagnostic and short-term prognosis value of serum ox-LDL/LOX-1 and GDF-15 in elderly patients with acute coronary syndrome

**DOI:** 10.3389/fmolb.2026.1811738

**Published:** 2026-05-08

**Authors:** Jinlin Wang, Tong Guo, Fengzhen Liu, Xueliang Pei, Fengxia Guo, Hanxiao Li, Facai Cui

**Affiliations:** 1 Department of Clinical Laboratory, Henan Provincial People’s Hospital, Zhengzhou, China; 2 Department of Cardiology, Xinxiang First People’s Hospital, Xinxiang, China; 3 Department of Cardiovascular Surgery, Fuwai Central China Cardiovascular Hospital, The Heart Center of Henan Provincial, Zhengzhou, China

**Keywords:** diagnosis, GDF-15, LOX-1, MACE, ox-LDL, short-term prognosis

## Abstract

**Background:**

In the diagnosis and short-term prognosis of elderly patients with acute coronary syndrome (ACS), there has been a persistent lack of effective biomarkers. This study aimed to investigate the expression characteristics of serum oxidized-low-density lipoprotein (ox-LDL), lectin-like-oxidized low-density-lipoprotein receptor-1 (LOX-1), and growth-differentiation-factor 15 (GDF-15) in elderly ACS patients and assess their short-term prognostic value.

**Methods:**

161 elderly patients with ACS and 66 elderly patients with non-ACS were enrolled. Subgroup classification included the ST-elevation myocardial infarction (STEMI), unstable-angina-pectoris (UAP) and NSTEMI. Serum ox-LDL, LOX-1 and GDF-15 were evaluated via ELISA assay. Receiver operating characteristic (ROC) curves were used to evaluate the diagnostic value. The prognosis of ACS patients was determined by Major-Adverse-Cardiovascular-Events (MACE) as the outcome. The short-term prognosis value of 3 serum biomarkers was evaluated using multivariate logistic analysis.

**Results:**

Ox-LDL, LOX-1, and GDF-15 levels in serum were significantly higher in elderly patients with ACS (all *P* < 0.001). The ROC analysis results showed that GDF-15 (AUC = 0.884) had high accuracy in diagnosing elderly patients with ACS. In subgroup analysis, serum levels of ox-LDL (*P* < 0.001) and LOX-1 (*P* < 0.05) were significantly lower in the UAP group than in the STEMI and NSTEMI groups. The APACHE II score (*P* = 0.005) and GDF-15 level (*P* < 0.001) were significantly higher in the MACE group. Multivariate logistic analyses further demonstrated that GDF-15 was an independent predictor of MACE in elderly ACS patients (*P* = 0.011).

**Conclusion:**

High serum GDF-15 levels are significantly associated with the presence of ACS and, in ACS patients, are significantly higher in those who do develop MACE. Serum GDF-15 can serve as an independent predictor of short-term MACE risk in elderly patients with ACS.

## Introduction

Acute coronary syndrome (ACS) is a critical condition characterized by sudden abnormalities in the blood supply to the heart. It typically results from a sudden blockage in the coronary arteries, presenting with sudden onset, rapid progression, and multiple complications (Nohria and Viera, 2024; Chapman et al., 2024; Bhatt et al., 2022). ACS includes a series of clinical entities, including ST-segment elevation myocardial infarction (STEMI), non-ST-segment elevation myocardial infarction (NSTEMI) and unstable angina pectoris (UAP). These entities have common underlying pathophysiological mechanisms, including atherosclerotic plaque rupture or erosion, but their clinical manifestations, management strategies and prognostic significance are different (Steg et al., 2012; Collet et al., 2021). Although several clinical scoring systems, such as the Gensini score, have been established to assess the risk stratification of ACS patients, these tools are often complex to operate in emergency settings and cannot fully reflect the molecular pathological mechanisms (Ben Halima et al., 2022; Atwood, 2022). On the other hand, compared to younger individuals when elderly people suffer from ACS, their symptoms are often “atypical”. Epidemiological data indicate that approximately 35%–40% of all ACS cases occur in older adults, and up to 44% of elderly patients with myocardial infarction do not report chest pain as their primary symptom, presenting instead with dyspnea, confusion, or gastrointestinal symptoms (Narendren et al., 2024; Birnbaum et al., 2012). These symptoms make the clinical diagnosis of elderly patients with ACS more challenging. At the same time, elderly ACS patients are more likely to develop complications, which pose a greater threat to survival (McGarry and Shenvi, 2021). Therefore, it is of great significance to explore the markers for diagnosis and treatment of ACS in elderly patients to improve the overall quality of life of ACS.

Although the early diagnosis and treatment of elderly ACS patients are more difficult, there is a lack of biomarkers for elderly ACS patients. Older adults exhibit age-related physiological changes, including increased prevalence of inflammaging, altered drug metabolism, and higher comorbidity burden, which can significantly impact biomarker performance and interpretation (Narendren et al., 2024; Moumneh et al., 2025). Nevertheless, elderly patients remain substantially underrepresented in major cardiovascular clinical trials, leading to a paucity of evidence-based guidelines tailored to this population (Narendren et al., 2024). Earlier studies found that myocardial markers serum levels were abnormal in older patients with more severe ACS (López-Lluva et al., 2014). A multicenter study demonstrated that frailty data was strongly associated with the risk of MACEs in elderly patients with non-ST-elevation acute coronary syndrome (NSTE-ACS) (van den Broek et al., 2024). Recent studies have also found that high uric acid levels are associated with the MACE outcomes of elderly patients with ACS (Rommers et al., 2025). Although these reports are helpful for diagnosis and treatment of elderly ACS patients. However, more novel biomarkers, especially serum markers, need to be explored.

With the rapid development of molecular biology and genomics, many markers related to the pathogenesis of ACS have been identified. Oxidized-low-density-lipoprotein (ox-LDL) promotes the progression of atherosclerotic lesions by inducing endothelial dysfunction and activating immune cells (Sohrabi et al., 2020; Khwaja et al., 2021). Its receptor, lectin-like-oxidized-low-density-lipoprotein receptor-1 (LOX-1), is widely recognized to be upregulated in vascular injury and plaque instability (Sharma et al., 2022; Sánchez-León et al., 2024). Thus, the ox-LDL/LOX-1 axis therefore reflects oxidative lipid modification and plaque instability, which are key mechanisms underlying the initiation of ACS (Sorokin et al., 2023). Additionally, Growth-differentiation-factor 15 (GDF-15), as a stress-related cytokine, reflects myocardial injury and systemic inflammatory status, and its elevated levels are closely associated with adverse cardiovascular events (Xiao et al., 2022; Silva-Bermudez et al., 2024; May et al., 2021). Unlike ox-LDL and LOX-1, which mainly reflect vascular atherosclerotic activity, GDF-15 represents the systemic stress and myocardial injury responses triggered during acute ischemic events (May et al., 2021). Therefore, these biomarkers originate from different but complementary biological pathways involved in ACS pathophysiology. Simultaneous evaluation of ox-LDL/LOX-1 and GDF-15 may provide a more comprehensive assessment of both atherosclerotic plaque activity and systemic myocardial stress. Although these biomarkers have been reported to be abnormally expressed in ACS patients, their combined diagnostic value and short-term prognostic significance in elderly ACS patients have not been fully elucidated.

Therefore, this study aimed to evaluate the diagnostic value of serum ox-LDL, LOX-1, and GDF-15 for ACS in elderly patients and to investigate their prognostic significance for short-term MACE. By comparing biomarker levels between elderly ACS patients and non-ACS controls, and between ACS patients with and without MACE, we sought to identify novel biomarkers that could improve risk stratification and guide clinical management in this high-risk population.

## Materials and methods

### Recruitment of participants information

This study enrolled 227 elderly patients who treated in the ED department at Henan-Provincial-People’s Hospital from September 2024 to March 2025. According to the 2020 standard of ACS, patients were grouped into an ACS group (n = 161) and a non-ACS control group (n = 66) (Collet et al., 2024). The ACS group was further subdivided into ST-segment-elevation-myocardial-infarction (STEMI, n = 50), NSTEMI, n = 79, and unstable angina pectoris (UAP, n = 32) groups. The non-ACS control group (n = 66) consisted of patients who presented with symptoms suggestive of myocardial ischemia (e.g., chest tightness and chest pain) but in whom ACS was ruled out based on serial electrocardiograms, cardiac troponin measurements, and clinical assessment. The final diagnoses in this group included stable angina pectoris, non-cardiac chest pain (e.g., musculoskeletal or gastrointestinal origin), and other cardiac conditions without acute ischemia (e.g., stable arrhythmias or well-compensated heart failure) (Mokhtari et al., 2015; Parkash et al., 2009). All patients signed informed consent, the research has been approved by the relevant departments of our unit (Approval number: 2024–168).

### Inclusion and exclusion criteria

The methods for inclusion and exclusion of ACS patients in this study refer to others (Parkash et al., 2009; Lukitasari et al., 2023; Kamali et al., 2014), as follows:

Inclusion criteria:

1. Age ≥60 years;
2. Complete clinical and auxiliary examination data available;
3. Underwent coronary angiography;
4. Patients presented with chest pain, chest tightness, and associated symptoms of myocardial ischemia.

Exclusion criteria:

1. Prior coronary intervention or coronary artery bypass grafting;
2. Structural heart diseases such as cardiomyopathy or valvular disease;
3. Estimated glomerular filtration rate (eGFR) < 60 mL/min/1.73 m^2^;
4. Presence of active infection or malignancy;
5. Autoimmune diseases;
6. Incomplete data.

The diagnosis of ACS was established according to current guideline recommendations of the European Society of Cardiology (Collet et al., 2021). The severity of coronary artery lesions was evaluated using the Gensini scoring system based on coronary angiography (Gensini, 1983).

### Clinical baseline indicators

Baseline demographic and clinical characteristics were recorded for all participants, including age, sex, history of hypertension, diabetes, smoking, coronary heart disease, and cerebral infarction. For patients in the ACS group, additional data were collected, including the number of diseased vessels (0, 1, 2, or multiple), presence of lesions in the right coronary artery (RCA), left anterior descending artery (LAD), and left-circumflex-artery (LCX), occurrence of MACE, ACS subtype (STEMI, NSTEMI, or UAP), Gensini score, and APACHE II score.

## Outcome index

The patients were followed up for 6 months after admission. The primary endpoint was in-hospital MACE, defined as composite events of all-cause death, recurrent myocardial infarction and unplanned revascularization during hospitalization (Cui et al., 2025). Recurrent myocardial infarction was defined according to the Fourth Universal Definition of Myocardial Infarction. Unplanned revascularization referred to any unscheduled percutaneous coronary intervention or coronary artery bypass grafting performed due to recurrent ischemia during the same hospitalization (Thygesen et al., 2018).

### Specimen collection and serum biomarker detection

Blood (4 mL) was collected from participants within 1 h of admission to the emergency department via serum tubes without anticoagulant. Samples were allowed to stand at room temperature for 2 h, centrifuged at 1000 *g* for 20 min, and serum was separated and stored at −80 °C. Serum creatine-kinase-MB (CK-MB) and high-sensitivity-troponin I (hs-TNI) were measured using VITROS ®5600 automatic biochemical immune analyzer (Catalog numbers: 6802413, Ortho Clinical Diagnostics, New Jersey, United States) following the manufacturer’s instructions. Serum ox-LDL was determined using the Human Ox-LDL Detection Kit (Catalog numbers: 20222400052, GOLDMAG, Xian, China). LOX-1 was measured by using the Human LOX-1 ELISA Kit (Catalog numbers: QT-EH0013, FineTest, Wuhan, China). GDF-15 levels were measured using the Human GDF-15 Detection Kit (Catalog numbers: 0524021, maccura, Chengdu, China).

### Imaging examination

Coronary angiography images were tested in a blinded fashion by two interventional cardiologists with over 5 years of experience, unaware of clinical grouping. LAD, LCX, and RCA categories are evaluated. Patients were classified by the number of diseased vessels: 0, 1, 2, or multi-vessel disease (≥3 vessels). The presence of lesions in RCA, LAD, or LCX was also recorded.

#### Scoring systems

Gensini Score: Coronary stenosis severity was scored as <25% (1+), 25%–50% (2+), 51%–75% (4+), 76%–90% (8+), 91%–99% (16+), Total occlusion (32+); multiplied by weighting coefficients based on lesion location (left main ×5, proximal LAD ×2.5, LCX/RCA ×1). APACHE II Score: Calculated based on patient age, acute physiological parameters and chronic health status.

### Statistical analysis

Kolmogorov (K-S) normality test was used to analyze the data. M (P25-P75) was used to show non-normal distribution data. The Kruskal–Wallis H test was used for comparison among multiple groups, and the Mann–Whitney U test was used for comparison between two groups. Categorical data were expressed as counts (percentages) and compared using the chi-square test. A receiver operating characteristic (ROC) curve was used to evaluate the ability of ACSs. Multivariate logistic regression models were applied to identify factors independently associated with short-term prognosis outcomes. Among them, the continuous variable GDF-15 is difficult to interpret clinically due to the small original unit magnitude. Referring to previous studies (Andersson et al., 2016), it was included in the multivariate logistic regression model after natural logarithm transformation. A *P*-value < 0.05 was considered statistically significant.

## Results

### Comparison of baseline characteristics between two study groups

**FIGURE 1:**
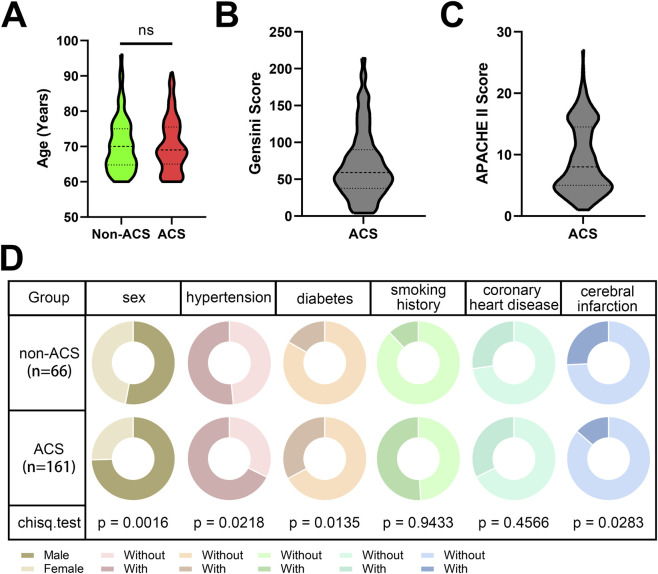
Comparison of Baseline Characteristics between Non-ACS and ACS group patients. **(A)** Age between Non-ACS and ACS group patients (analyzed by Mann-Whitney U test). **(B,C)** Gensini Score **(B)** and APACHE II Score **(C)** of ACS group patients. **(D)** Chi-square analysis of clinicopathological features in Non-ACS and ACS group patients. Ns represents no significant difference. ns, represents no significant difference.

First, to assess differences in clinical baseline characteristics between the two groups, we performed a comparative analysis (Table 1; Figures 1A–D). There were significant differences among Non-ACS and ACS groups in Gender, hypertension, diabetes, history of cerebral infarction (all *P* < 0.05, Table 1; Figure 1D). The proportion of males was significantly higher in the ACS group than in the Non-ACS group (120/161 vs. 35/66, *P* = 0.002). Similarly, the prevalence of hypertension (109/161 vs. 34/66, *P* = 0.022) and diabetes (53/161 vs. 11/66, *P* = 0.014) was markedly greater in patients with ACS. In addition, a history of cerebral infarction was more frequent in the ACS group compared with the Non-ACS group (22/161 vs. 17/66, *P* = 0.028). There were no significant differences in Age (median 69.00 vs. 70.00 years, *P* = 0.950), Smoking History (*P* = 0.943) and Coronary Heart Disease (*P* = 0.457). The baseline clinical and angiographic characteristics of the ACS group were as follows. Among the 161 ACS patients, multi-vessel disease (≥3 diseased vessels) was the most common, observed in 84 patients. Lesions in the RCA and LAD were each present in 143 patients, while LCX lesions were found in 104 patients. During hospitalization, MACE occurred in 131 patients. The distribution of ACS subtypes was as follows: STEMI in 50 patients, NSTEMI in 79 patients, and UAP in 32 patients. The median Gensini score was 59.00 (interquartile range: 37.50–90.00), and the median APACHE II score was 8.00 (5.00–14.50).

**TABLE 1:** Comparison of Baseline Characteristics between Non-ACS and ACS group patients.

Indicator	Non-ACS group (n = 66)	ACS group (n = 161)	*U* or *χ2* value	*P*
Age (years)	70.00 (64.75, 75.00)	69.00 (65.00, 75.50)	5285	0.950
Gender			9.995	0.002
Male	35	120		
Female	31	41		
Hypertension			5.261	0.022
No	32	52		
Yes	34	109		
Diabetes			6.108	0.014
No	55	108		
Yes	11	53		
Smoking history			0.005	0.943
No	58	79		
Yes	8	82		
Coronary heart disease			0.554	0.457
No	48	109		
Yes	18	52		
History of cerebral infarction			4.811	0.028
No	49	139		
Yes	17	22		
Number of diseased vessels			-	-
0 vessels	-	9		
1 vessel	-	36		
2 vessels	-	32		
Multiple vessels	-	84		
RCA lesion			-	-
No	-	55		
Yes	-	106		
LAD lesion			-	-
No	-	18		
Yes	-	143		
LCX lesion			-	-
No	-	57		
Yes	-	104		
MACE			-	-
No	-	30		
Yes	-	131		
Subtype			-	-
STEMI		50		
NSTEMI		79		
UAP		32		
Gensini score		59.00 (37.50, 90.00)	-	-
APACHE II score		8.00 (5.000, 14.50)	-	-

The Mann–Whitney U test was used for comparison between two groups (age). Chi-square analysis of clinicopathological features in Non-ACS, and ACS, group patients.

### Comparison of serum factors between Non-ACS and ACS group patients

**FIGURE 2:**
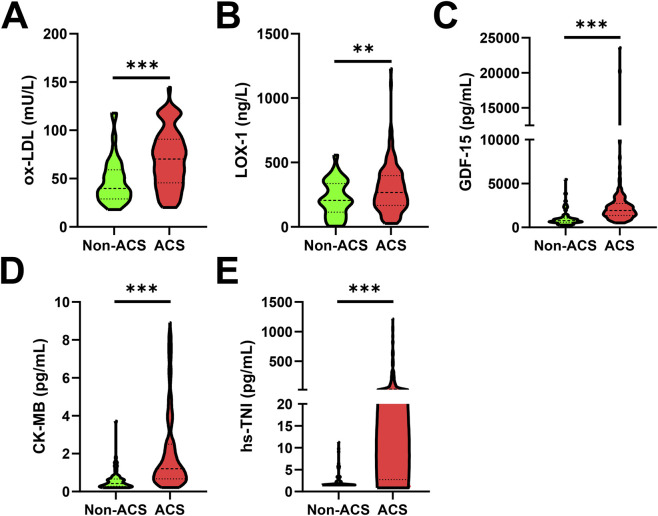
Comparison of 5 serum factors between Non-ACS and ACS group patients using the Mann-Whitney U test. **(A–E)** ox-LDL **(A)**, LOX-1 **(B)**, GDF-15 **(C)**, CK-MB **(D)** and hs-TNI **(E)** between Non-ACS and ACS group patients. **P < 0.01, ***P < 0.001.

Furthermore, to compare serum levels of the measured biomarkers between the non-ACS and ACS groups, we analyzed five serum factors, including ox-LDL, LOX-1, GDF-15, CK-MB, and hs-TNI (Figures 2A–E; Table 2). The levels of these 5 serum factors in the serum of the ACS group patients were significantly higher. Specifically, the median level of ox-LDL was markedly higher in ACS patients (70.22 mU/L) than in non-ACS patients (39.83 mU/L) (*P* < 0.001). Similarly, LOX-1 levels were increased in the ACS group (267.0 ng/L) compared with the non-ACS group (205.80 ng/L) (*P* = 0.009). GDF-15 was also significantly elevated in ACS patients (1923.00 pg/mL) relative to non-ACS patients (772.30 pg/mL) (P < 0.001). In addition, the classical myocardial injury markers CK-MB and hs-TNI were substantially higher in the ACS group (both P < 0.001).

**TABLE 2:** Comparison of serum factors between Non-ACS and ACS group patients using the Mann-Whitney U test.

Indicator	Non-ACS group (n = 66)	ACS group (n = 161)	*U* value	*P*
Ox-LDL (mU/L)	39.83 (28.90, 59.11)	70.22 (45.56, 90.82)	2,860	<0.001
LOX-1 (ng/L)	205.80 (114.40, 336.50)	267.0 (166.30, 398.30)	4,142	0.009
GDF-15 (pg/mL)	772.30 (580.10, 1,049.00)	1923.00 (1,355.00, 2,735.00)	1,656	<0.001
CK-MB (pg/mL)	0.42 (0.28, 0.66)	1.20 (0.67, 2.51)	1832	<0.001
Hs-TNI (pg/mL)	1.50 (1.50, 2.37)	28.28 (2.725, 147.20)	1,682	<0.001

### Analyze the diagnostic value of three serum factors for ACS

**FIGURE 3:**
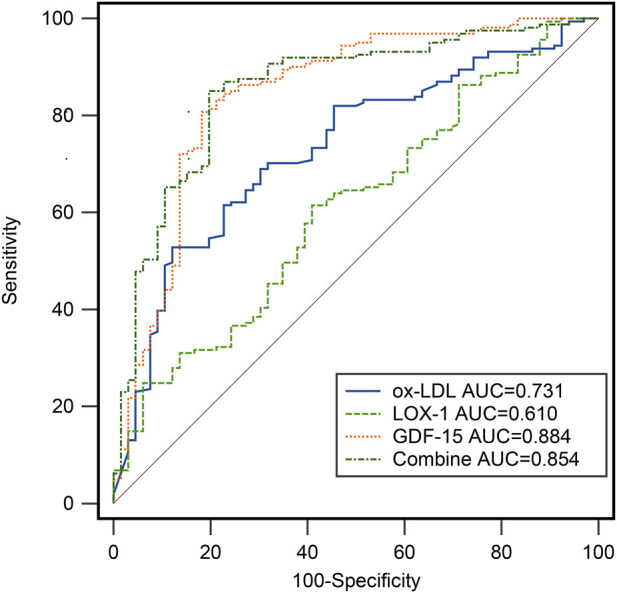
ROC analysis of 3 serum factors to distinguish Non-ACS and ACS patients.

To evaluate the diagnostic performance of ox-LDL, LOX-1, and GDF-15 for distinguishing between non-ACS and ACS patients, we conducted a ROC analysis (Figure 3; Table 3). The results showed that the AUC values of ox-LDL, LOX-1 and GDF-15 were 0.731, 0.610 and 0.884, respectively. Among these biomarkers, GDF-15 demonstrated the highest diagnostic performance. This suggests that serum GDF-15 may be a potential diagnostic marker for ACS.

**TABLE 3:** Analysis of diagnostic value of 3 factors alone or combination in patients with ACS.

Indicator	AUC	Specificity (%)	Sensitivity (%)	Youden index	95% CI
Ox-LDL (mU/L)	0.731	87.88	52.80	0.407	0.668–0.787
LOX-1 (ng/L)	0.610	59.09	61.49	0.206	0.543–0.674
GDF-15 (pg/mL)	0.884	81.82	80.75	0.569	0.790–0.889
Combine	0.854	80.30	85.09	0.654	0.801–0.897

### Subtype analysis of three serum factors in patients with ACS

**FIGURE 4:**
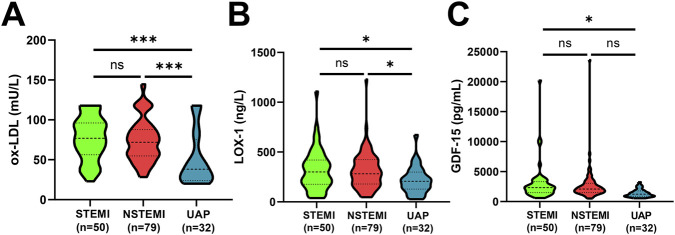
Comparison of 3 serum factors among STEMI, NSTEMI and UAP group patients using the Kruskal–Wallis H test. **(A–C)** ox-LDL **(A)**, LOX-1 **(B)**, GDF-15 **(C)** among STEMI, NSTEMI and UAP group patients. ns represents no significant difference, *P < 0.05, ***P < 0.001.

To investigate the expression levels of the three serum biomarkers across different ACS subtypes in elderly patients, we performed subgroup analyses based on ACS classification (STEMI, NSTEMI, and UAP) (Figures 4A–C). There was no significant difference in the levels of the three serum markers between patients with STEMI and those with NSTEMI (all *P* > 0.05, Figures 4A–C). The levels of the three serum indicators in the serum of STEMI patients were all higher than those of UAP patients (all *P* < 0.05, Figures 4A–C). In the comparison between NSTEMI and UAP, we found that the levels of ox-LDL and LOX-1 were significantly higher in NSTEMI group than in UAP group (all *P* < 0.05, Figures 4A,B).

### Analysis of the short-term prognostic value of three serum factors for patients with ACS

After analyzing the diagnostic value of the three serum factors, we further examined the correlation between these three indicators and the outcome of MACEs. First, we divided the ACS patients into two groups, and compared the baseline characteristics and five serum factor levels of the two groups (Table 4). The results showed that LCX lesion, subtype and APACHE II score were associated with whether the patients experienced MACE (all *P* < 0.05, Table 4). Furthermore, the levels of LOX-1 and GDF-15 were significantly higher in patients with MACE outcome compared to those without MACE (all *P* < 0.05, Table 4).

**TABLE 4:** Comparison of clinical data between ACS patients with MACE or without MACE.

Indicator	MACE (n = 131)	Without MACE (n = 30)	*U* or *χ2* value	*P*
Age (years)	69.00 (65.00, 75.00)	70.00 (62.75, 78.50)	1854	0.632
Gender			0.088	0.766
Male	97	23		
Female	34	7		
Hypertension			0.535	0.465
No	44	8		
Yes	87	22		
Diabetes			0.003	0.957
No	88	20		
Yes	43	10		
Smoking history			0.485	0.486
No	66	13		
Yes	65	17		
Coronary heart disease			1.355	0.244
No	86	23		
Yes	45	7		
History of cerebral infarction			0.282	0.596
No	114	25		
Yes	17	5		
Number of diseased vessels			6.103	0.107
0 vessels	8	1		
1 vessel	34	2		
2 vessels	25	7		
Multiple vessels	64	20		
RCA lesion			3.288	0.070
No	49	6		
Yes	82	24		
LAD lesion			0.756	0.385
No	16	2		
Yes	115	28		
LCX lesion			5.660	0.017
No	52	5		
Yes	79	25		
Subtype			20.850	<0.001
STEMI	31	19		
NSTEMI	68	11		
UAP	32	0		
Gensini score	55.00 (35.00, 85.00)	74.50 (51.50, 108.80)	01,520	0.053
APACHE II score	15.00 (5.75, 17.00)	7.00 (5.00, 12.00)	1,324	0.005
Ox-LDL (mU/L)	68.58 (42.88, 90.73)	75.85 (60.96, 91.81)	1,641	0.160
LOX-1 (ng/L)	349.80 (209.80, 507.00)	257.50 (158.20, 376.90)	1,473	0.032
GDF-15 (pg/mL)	2,530.00 (2033.39, 3,585.00)	1816.30 (1,276.77, 2,569.00)	1,148	<0.001
CK-MB (pg/mL)	1.20 (0.63, 2.48)	1.41 (0.65, 2.68)	1721	0.291
Hs-TNI (pg/mL)	28.28 (2.60, 148.10)	35.73 (3.46, 149.20)	1906	0.799

The Mann–Whitney U test was used for comparison between two groups (age). Chi-square analysis of clinicopathological features in Non-ACS, and ACS, group patients.

In order to further analyze the Independent predictive power of these indicators for patients with ACS, we conducted a multivariate analysis to examine the indicators with significant significance. The results showed that LCX lesion (OR = 1.793, *P* = 0.307), ACS subtype (OR < 0.001, *P* = 0.998), and APACHE II score (OR = 0.938, *P* = 0.126) were not independently associated with the occurrence of MACE. In contrast, GDF-15 remained significantly associated with MACE (OR = 0.360, *P* = 0.011), indicating that GDF-15 may serve as an independent predictor of adverse outcomes in patients with ACS (Table 5).

**TABLE 5:** Diagnosis factors of ACS patients with MACE by multivariate logistic analyses.

Characteristics	B	SE	Wald	HR (95%CI)	*P*
LCX lesion	0.503	0.581	0.749	1.653 (0.530–5.160)	0.387
Subtype	−18.447	6,944.755	0.000	0.000 (0.000–0.000)	0.998
APACHE II score	0.061	0.042	2.075	0.941 (0.867–1.022)	0.150
GDF-15	1.021	0.403	6.421	0.360 (0.163–0.793)	0.011

## Discussion

Age is an important risk factor for ACS and a key predictor of high mortality. It is estimated that the majority of STEMI occur in patients aged 65 years or older (Bhatt et al., 2022). In addition, the vast majority of myocardial infarction-related deaths occur in individuals aged 65 years of age or older (Sutton et al., 2023). Older patients with NSTEMI and STEMI are less likely to present with “typical” chest pain symptoms (Narendren et al., 2024). Therefore, identifying biomarkers for the diagnosis and prognosis of ACS in elderly patients is of great clinical significance. In this study, we explored the association between serum ox-LDL, LOX-1, and GDF-15 with ACS diagnosis and MACE outcomes, and found that these markers could serve as useful indicators.

Ox-LDL is a key pro-inflammatory factor in the development and progression of coronary atherosclerotic lesions. Studies have demonstrated that ox-LDL promotes endothelial cell injury, monocyte adhesion, and macrophage foam cell formation, thereby accelerating plaque formation and rupture (Yazdani et al., 2023). In this study, ox-LDL levels were significantly elevated in patients with STEMI and NSTEMI patients, a finding consistent with the extensive oxidative stress response characteristic of acute myocardial infarction. Previous literature has indicated that LOX-1, as the primary receptor for ox-LDL, mediates oxidative stress and inflammatory signaling, inducing endothelial dysfunction and apoptosis (Alieva et al., 2025). Rasodli et al. confirmed that LOX-1 expression is significantly upregulated in acute coronary events and is associated with plaque instability (Kłosowicz et al., 2024). In the present study, LOX-1 was also significantly elevated in the STEMI and NSTEMI groups and was positively correlated with GDF-15 levels, further suggesting its involvement in the myocardial injury and inflammatory pathological processes.

GDF-15 is a stress-response protein that reflects oxidative stress and inflammatory levels in the body (Sygitowicz et al., 2021). Studies have shown that elevated GDF-15 levels are associated with the rupture and instability of coronary atherosclerotic plaques, serving as an important biomarker for predicting poor prognosis in ACS patients (Lyu et al., 2024). Our results demonstrated that GDF-15 levels were significantly elevated in ACS patients. Mechanistically, GDF-15 may play a dual role in coronary lesion repair and progression of coronary lesions by regulating macrophage polarization and suppressing excessive inflammatory responses (Netala et al., 2024). Additionally, elevated GDF-15 may promote vascular smooth muscle cell apoptosis, leading to reduced stability and an increased risk of acute coronary events (Kłosowicz et al., 2024). This finding is consistent with the pathogenic mechanisms of GDF-15 in cardiovascular diseases reported by Wollert et al. (Khwaja et al., 2021).

Evaluating MACE in ACS is of great significance for the prognosis management of patients, the adjustment of treatment strategies, and risk stratification. In recent years, several markers for predicting MACE have been identified and reported (Liang et al., 2023). Han et al. found that the systemic inflammatory response index (SIRI) was closely associated with MACE outcomes in patients with ACS (Han et al., 2022). Other researchers have also found that in patients hospitalized for ACS, TACE/ADAM17 had a relatively high accuracy in predicting MACE (Chemaly et al., 2022). Our previous research also showed that compared with traditional biomarkers, copeptin serve as an independent predictor of MACE in patients with NSTEMI (Cui et al., 2025). However, there remains a relative lack of research on predictive markers for MACE in elderly patients with ACS. In the present study, we analyzed three serum markers in patients with and without MACE and performed multivariate logistic regression analysis. We observed that patients with MACE had higher GDF-15 levels in univariate analysis, whereas multivariate analysis identified GDF-15 as an independent predictor. This apparent inconsistency may be explained by the influence of confounding clinical variables. In ACS patients, biomarkers such as GDF-15 can be affected by disease severity, comorbidities, and systemic inflammatory status. After adjusting factors such as coronary artery lesion characteristics and APACHE II score, the independent predictive association between GDF-15 and MACE became evident. Consistent with the therapeutic mechanism of GDF-15 in cardiovascular diseases discussed in the previous section, the relatively low circulating GDF-15 levels in patients with MACE may reflect an insufficient protective stress response during the acute phase of myocardial injury. Taken together, our results suggest that GDF-15 may provide additional prognostic information regarding adverse cardiovascular outcomes in ACS patients, although the effect size is the regression coefficient is moderate due to measurement scale. These findings have several potential clinical implications. First, the high diagnostic accuracy of GDF-15 for ACS in elderly patients supports its potential as an emergency auxiliary biomarker, particularly for patients with atypical manifestations. Second, the prognostic value of GDF-15 for short-term MACE in elderly ACS patients may help improve risk stratification and guide clinical decision-making regarding monitoring intensity and treatment strategies. Third, this study supports the development of age-specific biomarkers in clinical practice by focusing on a population that is often underrepresented in elderly-cardiovascular studies. There are also some shortcomings of this study that need to be acknowledged. First, this is a single-center study with modest sample size and no external validation cohort. We explored the association of three serum markers with ACS using patients from a single center; while this design is acceptable for an exploratory analysis, it inherently limits the generalizability of our findings. This is particularly relevant given the heterogeneity of clinical presentations, comorbidities, and outcomes in the elderly ACS population. Therefore, the results require validation in larger, multicenter cohorts. Second, this study did not identify biomarkers that can accurately distinguish between the different subtypes of ACS, which represents an important direction for future research. Third, as an exploratory study, no formal prior sample size calculation was performed. Finally, although we observed an association between the biomarkers and clinical outcomes, we cannot determine whether these biomarkers directly affect pathophysiology or merely reflect underlying disease processes; this warrants further investigation in cell and animal models.

This study investigated the association of three serum markers with the occurrence and short-term prognosis of ACS in elderly patients. Serum GDF-15 demonstrated potential utility in distinguishing ACS from non-ACS patients. Serum levels of ox-LDL, LOX-1, and GDF-15 were significantly lower in UAP patients compared to those with STEMI or NSTEMI. Furthermore, GDF-15 was identified as an independent predictor of MACE in ACS patients. Collectively, these serum markers offer a new perspective for improving the early diagnosis and rational treatment of ACS patients.

## Data Availability

The original contributions presented in the study are included in the article/supplementary material, further inquiries can be directed to the corresponding author.
